# Exposure of Human CD4 T Cells to IL-12 Results in Enhanced TCR-Induced Cytokine Production, Altered TCR Signaling, and Increased Oxidative Metabolism

**DOI:** 10.1371/journal.pone.0157175

**Published:** 2016-06-09

**Authors:** Aldo Vacaflores, Nicole M. Chapman, John T. Harty, Martin J. Richer, Jon C. D. Houtman

**Affiliations:** 1 Interdisciplinary Graduate Program in Immunology, University of Iowa, Iowa City, Iowa, United States of America; 2 Department of Microbiology, Carver College of Medicine, University of Iowa, Iowa City, Iowa, United States of America; 3 Department of Pathology, University of Iowa, Iowa City, Iowa, United States of America; 4 Department of Internal Medicine, Division of Immunology, University of Iowa, Iowa City, Iowa, United States of America; J. Heyrovsky Institute of Physical Chemistry, CZECH REPUBLIC

## Abstract

Human CD4 T cells are constantly exposed to IL-12 during infections and certain autoimmune disorders. The current paradigm is that IL-12 promotes the differentiation of naïve CD4 T cells into Th1 cells, but recent studies suggest IL-12 may play a more complex role in T cell biology. We examined if exposure to IL-12 alters human CD4 T cell responses to subsequent TCR stimulation. We found that IL-12 pretreatment increased TCR-induced IFN-γ, TNF-α, IL-13, IL-4 and IL-10 production. This suggests that prior exposure to IL-12 potentiates the TCR-induced release of a range of cytokines. We observed that IL-12 mediated its effects through both transcriptional and post-transcriptional mechanisms. IL-12 pretreatment increased the phosphorylation of AKT, p38 and LCK following TCR stimulation without altering other TCR signaling molecules, potentially mediating the increase in transcription of cytokines. In addition, the IL-12-mediated enhancement of cytokines that are not transcriptionally regulated was partially driven by increased oxidative metabolism. Our data uncover a novel function of IL-12 in human CD4 T cells; specifically, it enhances the release of a range of cytokines potentially by altering TCR signaling pathways and by enhancing oxidative metabolism.

## Introduction

Upon encounter with cognate antigen presented by antigen presenting cells, naïve CD4 T cells proliferate and differentiate into effector and memory subsets. These activated (effector or memory) CD4 T cells then recirculate through various tissues to aid infection clearance and protect against pathogen re-exposure. Emerging literature suggests that T cell differentiation occurs in several steps. Initially, naïve and central memory CD4 T cells are primed in secondary lymphoid organs. These primed but not fully differentiated cells are released from the secondary lymphoid organs and migrate to the sites of inflammation. Full CD4 T cell differentiation then occurs at sites of infection and inflammation after they have migrated from the secondary lymphoid organs [1]. Furthermore, it is becoming increasingly clear that the activated CD4 T cells are not all terminally differentiated and the majority can actually remain flexible and be re-polarized in different inflammatory environments [2–5]. Therefore, it is clear that the myriad of inflammatory signals that effector and memory CD4 T cells receive from the environment play key roles in influencing their subsequent responses.

One of the signals controlling CD4 T cell function is the inflammatory cytokine IL-12. This cytokine is produced in response to pathogens, but is also present at inflammatory sites in human disorders [6–10]. The current paradigm is that IL-12 primarily promotes the differentiation of naïve CD4 T cells into Th1 cells [9]. Also, the presence of IL-12 during priming of naïve CD8 T cells has been shown to promote strong effector functions and memory development [11]. Interestingly, the majority of these studies have focused on the effects of IL-12 if it is present during priming of naïve T cells (IL-12 present before or following TCR stimulation). However, after the initial priming, activated CD4 T cells that are not fully differentiated will also be exposed to IL-12 as they migrate through the circulatory or lymphatic system before entering the sites of infection, where they will be further activated via TCR induction by antigen presenting cells. The effects of IL-12 signals in altering the response of activated, non-fully differentiated T cells to subsequent TCR stimulation remains poorly understood. Recent murine studies, suggest that IL-12 alters the responses of activated T cells to subsequent TCR stimulation, suggesting that the modulation of CD4 T cell responses by IL-12 is more complex than simply inducing the differentiation of Th1 cells. In this regard, Richer et al. and Kim et al. demonstrated that murine effector/memory CD8 or secondary effector CD4 T cells exposed to *in vivo* inflammatory signals, driven primarily by IL-12 and/or type I interferons, have an altered response to *ex vivo* re-challenge with antigen [12, 13]. In these studies, exposure to IL-12 decreased the dose of antigen required to stimulate the maximal T cell response (also known as functional avidity). Likewise, murine memory CD8 T cells conditioned with IL-12 and IL-18 *in vitro* have enhanced cytokine production and cytotoxic activity upon TCR re-stimulation [14]. In addition, previous studies from us and others has demonstrated that prior exposure to IL-7, IL-15 or a TLR5 ligand increases the responsiveness of human T cells to TCR stimulation [15, 16]. Collectively these studies suggest that prior exposure to different cytokines or inflammatory signals alters how T cells respond to TCR stimulation.

Although these studies provide insight into murine T cell biology, whether IL-12 similarly regulates the function of human T cells and the precise molecular mechanism by which IL-12 alters subsequent TCR-mediated responses has not been fully elucidated. To explore these questions we used a system consisting of human peripheral blood CD4 T cells that have been activated under non polarizing conditions, which models primed, but not fully differentiated, human CD4 T cells that are released from the secondary lymphoid organs into circulation. We found that prior exposure to IL-12 elevated the response of human activated CD4 T cells to stimulation via the TCR. The IL-12 mediated increases in responses to TCR stimulation seemed to be mediated by two distinct mechanisms: increased activation of select TCR signaling molecules and increased metabolic respiration. This data suggest that the regulation of CD4 T cell function by IL-12 is more complex than simply driving Th1 differentiation. Instead it seems that IL-12 is continually shaping human CD4 T cell responses in a context-specific manner. Based on our results we propose a model in which IL-12 present in blood, infection sites, and/or at inflammatory sites primes human effector or memory CD4 T cells that are not terminally differentiated, allowing them to respond faster when they encounter their cognate antigen at sites of infection and be more easily polarized depending upon the cytokine milieu they encounter.

## Materials and Methods

### Human samples

Peripheral blood mononuclear cells (PBMCs) were obtained from anonymous donors as previously described [17]. Blood donors at the DeGowin Blood Center at the University of Iowa Hospitals and Clinics between 18 and 55 years provided written informed consent for cells not used for transfusion to be used for research. The consent process was approved by the University of Iowa’s Institutional Review Board. The signed written consent forms are maintained by the DeGowin Blood Center. The completely deidentified samples were then provided to investigators at the University of Iowa. Because all cells were obtained from discarded products, the donors approved for the research use of their cells, and the donors were de-identified, we did not required further Institutional Review Board approval to use these blood samples. All human subject studies were in compliance with the Declaration of Helsinki.

### Isolation and cytokine pretreatment of human activated CD4 T cells

CD4 T cells were negatively selected from PBMCs using the human CD4 T cell enrichment kit (Stem cell Technologies) to provide >98% CD4 T cells (data not shown). The cells were activated for 5 days with bead-bound anti-TCR/CD28 antibodies in the presence of IL-2. This method results in cells that are 100% positive for CD4 and 90–96% positive for CD45RO [18]. The cells were rested without stimulation for 24 h in complete RPMI (RPMI 1640 with 10% FBS, penicillin/streptomycin, and l-glutamine). The activated CD4 T cells were then treated with or without recombinant cytokines (R&D Systems) for different times and doses in complete RPMI. After pretreatment, cells were washed with RPMI 1640 three times to remove the cytokines.

### Cytokine production measured by ELISA

After cytokine pretreatments, cells were resuspended in complete RPMI and stimulated with plate-bound anti-TCR (anti-CD3 OKT3 clone)for 24 h. For the rest experiments, pretreated cells were rested for various times before anti-TCR stimulation (anti-CD3 OKT3 clone). For the oligomycin inhibitor experiment, pretreated cells were TCR-stimulated (anti-CD3 OKT3 clone) in the presence of oligomycin (2.5 μM). For the inhibition of glycolysis experiment, pretreated cells were TCR-stimulated (anti-CD3 OKT3 clone) in the presence of 2-DG (5 mM). Cytokine release was measured in triplicate using standard TMB based ELISA. To account for the variations between human donors and the conditions of independent experiments, data were normalized for each donor as: Fold-increase over no cytokine = (concentration of treated sample ÷ maximum concentration of no cytokine sample). For functional avidity, data were normalized as: Percent maximum response = (concentration of sample ÷ maximum concentration of respective treatment) x 100%. Normalized data was fitted to a sigmoidal curve to calculate EC_50_. However, absolute values obtained from the ELISAs are shown in S1, S3, S5 and S6 Figs as a reference.

### Intracellular cytokine staining and flow cytometry

For intracellular staining, pretreated cells were resuspended in complete RPMI and stimulated with 6 μg/mL of plate-bound anti-TCR (anti-CD3 OKT3 clone) for 8, 18, and or 24 h, in the presence or absence of brefeldin A or Monensin (BioLegend) added for the last 6 h. Cells were washed in FACS buffer (PBS, 10% FBS, and 0.05% sodium azide), and then stained for cytokines per manufacturer’s suggestion (Biolegend). All antibodies were purchased from Biolegend. For surface molecule staining, pretreated cells were washed in FACS buffer and stained on ice with the primary and/or secondary antibodies, followed by FACS analysis. Live lymphocytes were gated based on forward and side scatter. Quadrants were set so the baseline cytokine production of non-TCR stimulated cells was less than 1%. For survival assays, pretreated cells were washed in Annexin V binding buffer and stained with annexin V and propidium iodine (PI) per manufacturer’s suggestion (BD Biosciences). Annexin V and PI double negative cells were considered as viable cells, annexin V positive but PI negative cells as apoptosis undergoing cells, and annexin V and PI double positive cells as dead cells.

### Quantitative Real-time PCR

After cytokine pretreatments, cells were stimulated with 6 μg/mL of plate-bound anti-TCR (anti-CD3 OKT3 clone) for 8 and 18 h. cDNA was synthesized from total RNA and real-time RT-PCR was performed on an Applied Biosystems Model 7000 using SYBR Green. The expression of mRNA was normalized to that of mRNA encoding β-actin and quantification of fold induction of treated *vs* untreated was analyzed by the 2^-Δ ΔCT^ method [19].

### Immunoblotting

Activated CD4 T cells were treated with IL-12 (50 ng/mL) for different times and immunoblotting was performed as described previously [20, 21]. Alternatively IL-12 pretreated cells were stimulated with 2 μg/mL of crosslinked anti-TCR (anti-CD3 clone OKT3) and anti-CD4 (clone RPA-T4) and immunoblotting was performed. We use anti-TCR in combination with anti-CD4 because this provides the best enhancement of proximal and distal TCR signaling [20, 22, 23]. The intensity of the immunoblotting bands was determined using the Licor Odyssey v3.0 software. Normalization of the phospho-protein intensity to the actin intensity was conducted as described previously [24–27].

### Antibodies

The following antibodies were used for immunoblotting, cell-surface, and intracellular stains: The anti-LAT Y191 and anti-LAT from Millipore. The anti-LCK pY505, and anti-SLP-76 pY128, anti-IL-12Rβ1, and annexin V from BD Biosciences. The anti-PLC-γ1 pY783, anti-p38 pT180/Y182, anti-AKT pT308, anti-ZAP-70 pY319, anti-SRC pY416, anti-PLC-γ1, anti-LCK, anti-SLP-76, anti-AKT, anti-STAT4, anti-STAT4 pY693, anti-CD25, and anti-ERK 1/2 antibodies were purchased from Cell Signaling Technologies. The anti ERK1/2 pTpY185/187 and anti-AKT pS473 were from Invitrogen. The anti-GAPDH was from Meridian Life Science. The DyLight 800 and DyLight 680 labeled secondary antibodies were obtained from Thermo Scientific. The FITC anti-IFN-γ (4S.B3), Alexa Fluor 647 anti-IL-10 (JES3-9D7), Alexa Fluor 647 anti-IL-4 (8D4-8), Alexa Fluor 647 anti-TNF-α (Mab11), and APC anti-IL-13 (JES10-5A2) all from BioLegend. The anti-CD3 (OKT3), anti-CD4 (RPA-T4), FITC anti-CD28 (CD28.2), anti-CD-2 (RPA-2.10), anti-CD49d (9F10), anti-CD11a (HI111), FITC anti-CD278, anti-CD150, and DyLight 488 IgG (Ply4053) antibodies and propidium iodine were obtained from Biolegend. Anti-IL-12 Rβ2 was purchased from R&D Systems.

### Seahorse and ECAR/OCR measurements

After cytokine pretreatments, cells were resuspended in XF media (DMEM with glucose, pyruvate and glutamine) and were plated onto poly-L-lysine-coated XF-96 plates. Cells were then incubated for 30 min in a non-CO_2_ incubator and their metabolic profiles examined using an XF-96 Extracellular Flux Analyzer (Seahorse Bioscience). The optimal concentrations of mitochondrial inhibitors were Oligomycin 2.5 μM, FCCP 1.5 μM, Rotenone 5 μM and Antimycin A 5 μM (data not shown). OCR and ECAR values were normalized to cell numbers as previously described [28]. Metabolic parameters were then assessed as described before [29] using: (1) Basal respiration = (Basal OCR before adding Oligomycin)–(OCR following Rotenone/Antimycin A); (2) Maximal respiratory capacity = (OCR peak rate following FCCP)–(OCR following Rotenone/Antimycin A); (3) Basal ECAR = Basal ECAR rate before adding Oligomycin; and (4) Maximal ECAR = ECAR rate following addition of Rotenone/Antimycin A.

### Glucose uptake assay

After cytokine pretreatment, cells were washed in glucose free-medium and then cells were resuspended in glucose free medium containing 2-NBDG for 40 minutes at 37°C. Cells were then washed and analyzed by flow cytometry.

### Statistical analysis

Statistical analysis between the groups was assessed using GraphPad Prism. Specific tests for statistical significance are indicated in the figure legends. Differences were considered significant when p values were below 0.05.

## Results

### Prior exposure to IL-12 alters human activated CD4 T cell responses to subsequent TCR stimulation

To address if prior exposure to inflammatory cytokines altered how human activated CD4 T cells respond to TCR stimulation, primary human CD4 T cells were isolated from PBMCs and then activated for 5 days under non-polarizing conditions with anti-TCR/CD28 antibodies and recombinant IL-2. Following stimulation, cells were rested without stimulatory signals for 24 h before further analysis of downstream functions. Our system is intended to mimic the *in vivo* situation of a primed, but not fully differentiated, human CD4 T cell entering a site of infection, where it will encounter inflammatory signals and antigen. Activated CD4 T cells were then incubated with recombinant cytokines, extensively washed to remove the cytokines and stimulated with plate-bound anti-TCR antibodies (anti-CD3 clone OKT3). No additional costimulatory signals were provided to focus exclusively on the effects of the inflammatory cytokine on TCR-induced T cell activation.

**Fig 1:**
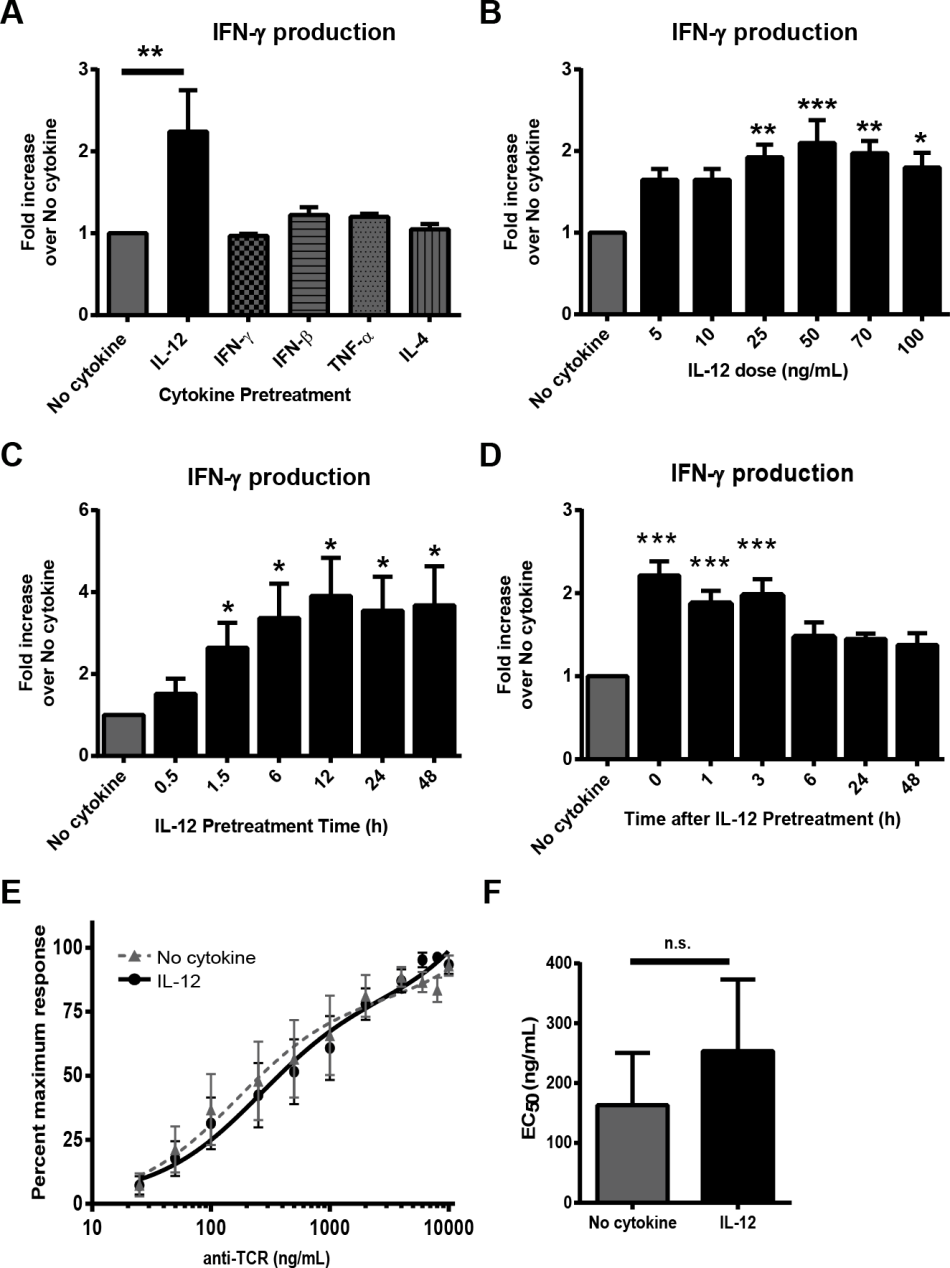
Exposure to IL-12 selectively alters how human activated CD4 T cells respond to TCR stimulation. Human activated CD4 T cells were left untreated (no cytokine) or treated (A) for 6 h with various cytokines (50 ng/mL), (B) various doses of IL-12, or (C) various times with 50 ng/mL of IL-12. (A-C) After pretreatment, cells were washed and stimulated with 2 μg/mL of plate bound anti-TCR antibodies (anti-CD3 clone OKT3) for 24 h. (D) Human activated CD4 T cells were pretreated for 6 h with 50 ng/mL of IL-12, rested for various times and then stimulated with plate bound anti-TCR antibodies (2 μg/mL) (anti-CD3 clone OKT3) for 24 h. IFN-γ production was determined by ELISA and results were normalized to the amounts produced by no cytokine. Graphs are shown as the mean ±SEM of normalized values from four to seven different donors. (E and F) Human activated CD4 T cells were incubated with or without 50 ng/ml IL-12 for 6 h and then stimulated with titrating doses of plate bound anti-TCR antibodies (anti-CD3 clone OKT3) for 24 h. The IFN-γ production was determined by ELISA. Data from five separate donors were normalized, plotted using GraphPad Prism and the EC_50_ values calculated. Data in (A), (B), and (D) was statistically compared to no cytokine cells using an unpaired one-way ANOVA. Data in (C) and (F) were statistically compared to no cytokine cells with a two-tail, unpaired Student’s t test. *p<0.05; **p<0.01; ***p<0.001; n.s. or no symbol = not significant.

Pretreatment with IFN-γ, IFN-β, TNF-α, or IL-4 had little to no effect on TCR-induced IFN-γ production in comparison to cells treated in media alone (Fig 1A, S1 Fig). In contrast, pretreatment with IL-12 significantly enhanced the TCR-induced production of IFN-γ compared to untreated cells, but did not cause measurable IFN-γ production in the absence of TCR stimulation (Fig 1A, S1 Fig). No synergistic or antagonist effects on IFN-γ production were seen when IL-12 was used in combination with IFN-γ, IFN-β, TNF-α, or IL-4 (data not shown). It was possible that the increased release of IFN-γ in the IL-12 pretreated group was due to differences in cell numbers after TCR stimulation. However, 24 hours after TCR stimulation cells stimulated with or without IL-12 had similar cell numbers (S2 Fig), suggesting that IL-12 did not alter proliferation in the timeframe of these experiments. In addition, we examined if IL-12 pretreatment altered the susceptibility to cell death before or after TCR stimulation as assessed by annexin V and PI staining. We found that before and after TCR stimulation both IL-12 pretreated and untreated cells have similar numbers of apoptotic cells (S2 Fig). Finally, we explored the status of CD25 on the IL-12 pretreated and untreated cells by flow cytometry. Both IL-12 pretreated and untreated have similar expression of CD25 (S2 Fig). Together, these data show that prior exposure to IL-12 selectively enhances human activated CD4 T cells to produce more IFN-γ after TCR stimulation.

To more fully characterize these effects, the optimal dose of IL-12 that increases TCR-induced IFN-γ production was determined. To test for this, human activated CD4 T cells were pretreated for 6 h with various doses of IL-12 and then stimulated with plate-bound anti-TCR antibodies (anti-CD3 clone OKT3). Pretreatment of cells with doses of 25–100 ng/mL of IL-12 significantly augmented IFN-γ production in comparison to cells with no IL-12 pretreatment (Fig 1B and S1 Fig). Interestingly, the doses of IL-12 that altered the responses of human activated CD4 T cells to TCR stimulation (25–100 ng/mL) are similar to the doses used in many other in vitro studies [14, 30–33] but they are above the concentrations previously described in serum of humans [34–37]. Next, the duration of IL-12 pretreatment required to increase IFN-γ production was examined. As shown in Fig 1C and S1 Fig, exposure to IL-12 for at least 1.5 hours was required to significantly increase the production of IFN-γ in comparison to cells with no IL-12 pretreatment. This finding suggests that the effects of IL-12 are not due to the presence of residual cytokine during TCR stimulation, since alter functions from residual IL-12 would be observed at all pretreatment times. Finally, we examined the duration of the effects after the removal of IL-12. To accomplish this, activated CD4 T cells were rested for various times after treatment with IL-12 before then being stimulated through the TCR (anti-CD3 clone OKT3). As shown in Fig 1D and S1 Fig, the ability of IL-12 to enhance IFN-γ production following TCR stimulation was short-lived and lasted for 3–6 hours after cessation of IL-12 treatment. In previous studies examining murine effector/memory CD8 T cells and secondary effector CD4 T cells, inflammatory cytokines decreased the dose of peptide antigen required to stimulate the maximal T cell response, known as functional avidity [12, 13]. Therefore, we examined if IL-12 pretreatment will have similar effects in human activated CD4 T cells using an antibody based stimulation. To this end, IL-12 pretreated cells were stimulated with titrating doses of plate bound anti-TCR antibodies (anti-CD3 clone OKT3) and then the functional avidity was determined by calculating the dose of stimulatory antibody needed to induce 50% of maximal IFN-γ production (EC_50_). We found that IL-12 exposure does not alter the functional avidity of human activated CD4 T cells in response to TCR ligation alone (Fig 1E and 1F). Collectively, these data demonstrate that IL-12 pretreatment transiently increases TCR-induced IFN-γ production after short exposure with IL-12. Furthermore, in an antibody based stimulation system, IL-12 exposure did not alter the functional avidity of human activated CD4 T cells in response to TCR ligation.

### Pretreatment of human activated CD4 T cell with IL-12 primes the TCR-induced release of a range of cytokines

**Fig 2:**
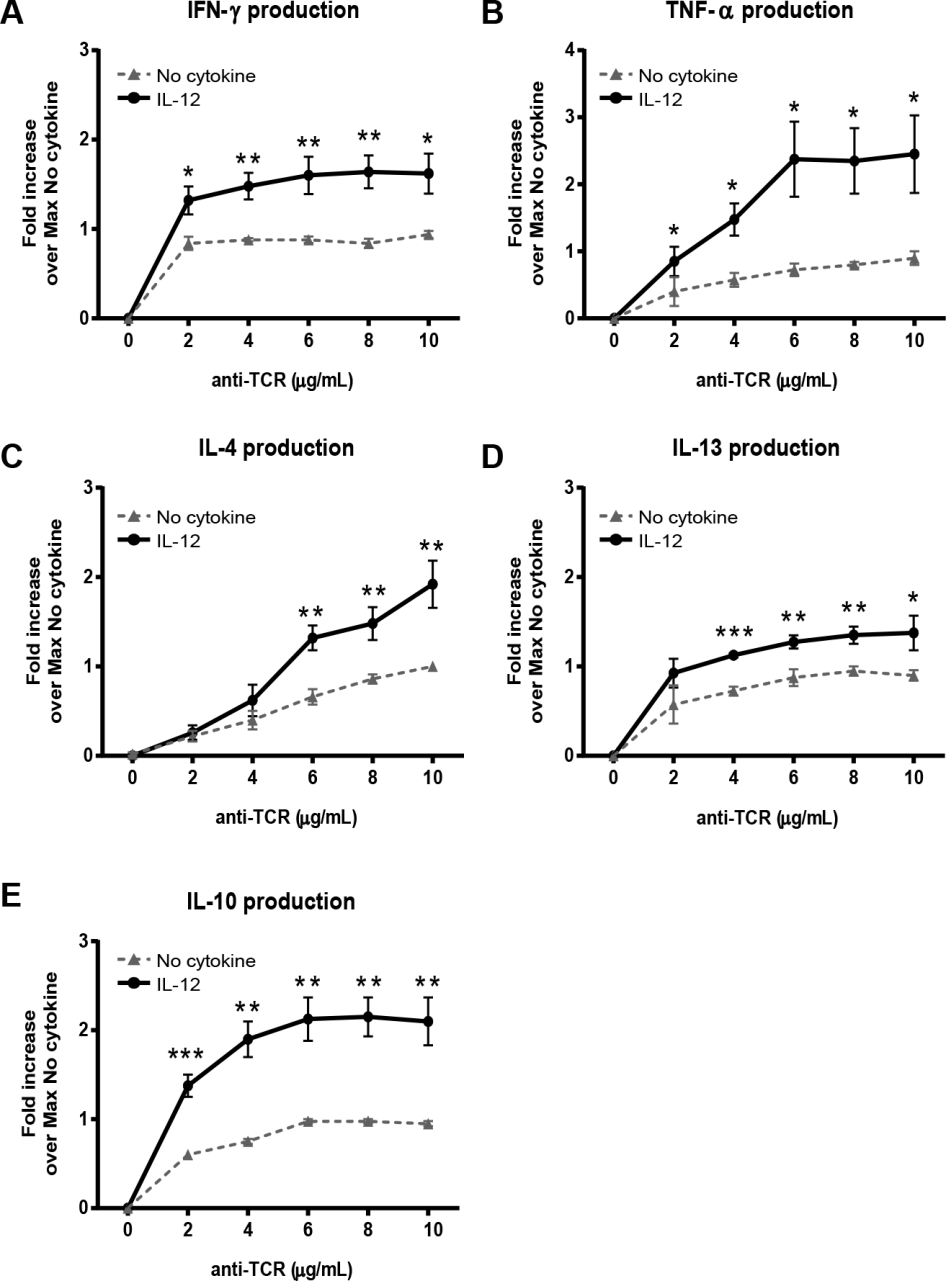
IL-12 pretreated human CD4 T cells have enhanced production of a range of cytokines following TCR stimulation. Human activated CD4 T cells were left untreated or pretreated with 50 ng/mL of IL-12 for 6 h and then subsequently stimulated with various doses of plate bound anti-TCR antibodies (anti-CD3 clone OKT3) for 24 h. Protein levels of (A) IFN-γ, (B) TNF-α, (C) IL-4, (D) IL-13, and (E) IL-10 were assessed by ELISA. Data for each treatment in an individual experiment were normalized to the maximum amount produced by no cytokine cells. The mean normalized value ± SEM from four to five different donors is shown. Data was analyzed with two-tail, unpaired Student’s t test. *p<0.05; **p<0.01; ***p<0.001; n.s. = not significant.

CD4 T cells have the capacity to produce multiple cytokines, including IFN-γ, TNF-α, IL-4, IL-13, and IL-10 [3, 38]. Numerous studies have established that IL-12 induces naive CD4 T cell differentiation into IFN-γ and TNF-α-producing Th1 subsets [39]. Therefore, we examined whether prior exposure to IL-12 was priming CD4 T cells to selectively produce Th1 cytokines or generally upregulate cytokine production. We observed that TCR engagement led to the production of IFN-γ, TNF-α, IL-4, IL-13, and IL-10 in cells pretreated with media alone (S3 Fig). Prior exposure to IL-12 not only increased the TCR-induced production of IFN-γ, but also enhanced the release of TNF-α, IL-4, IL-13 and IL-10 cytokines (Fig 2A–2E and S3 Fig). This IL-12-mediated potentiation of cytokine production was statistically significant for all cytokines at TCR doses of 6 μg/mL and higher (Fig 2A–2E). These data indicate that IL-12 pretreatment in activated human CD4 T cells does not solely increase Th1 cytokines, but instead enhances the production of a range of cytokines following TCR stimulation.

**Fig 3:**
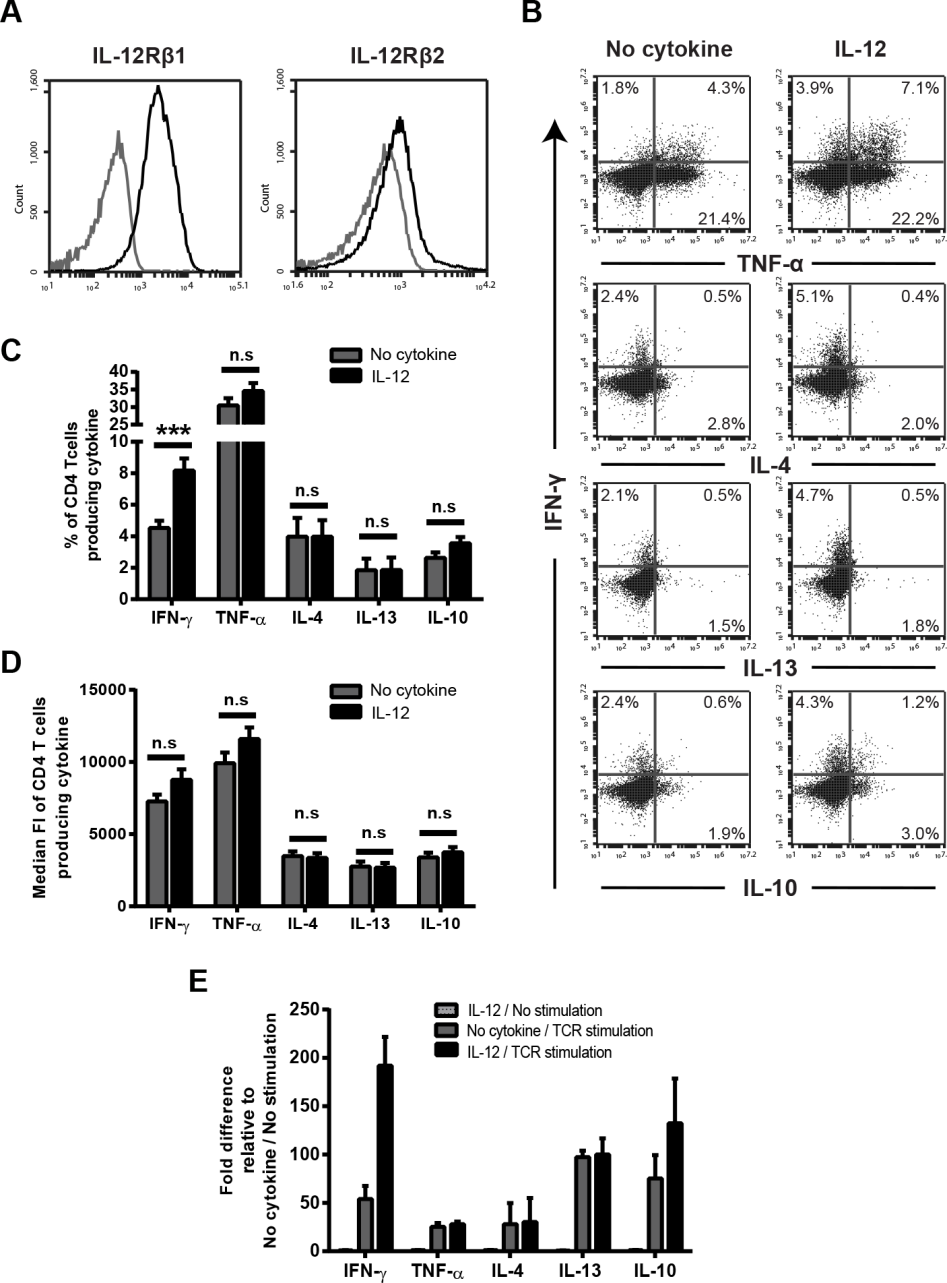
IL-12 mediated enhancement of cytokine production following TCR stimulation is mediated by a function of both transcriptional and post-transcriptional effects. (A) IL-12R β1 and IL-12R β2 expression was measured on human activated CD4 T cells by flow cytometry. Black line represents staining with IL-12R antibody and gray line represents staining with isotype-matched control antibody. Blots are representative of five different donors. (B-D) Human activated CD4 T cells were left untreated or pretreated with 50 ng/mL IL-12 for 6 h and then subsequently stimulated with 6 μg/mL plate bound anti-TCR antibodies (anti-CD3 clone OKT3) for 18 h. Protein levels of IFN-γ, TNF-α, IL-4, IL-13, and IL-10 were determined by analysis of intracellular-cytokine staining. Live lymphocytes were gated based on forward and side scatter. Quadrants were set so the baseline cytokine production of non-TCR stimulated cells was less than 1%. The frequencies and median fluoresce intensities of cytokine expression were then determined. Data are shown as (B) representative plots or (C and D) mean ± SEM of five to nine separate donors. (E) Gene expression of IFN-γ, TNF-α, IL-4, IL-13 and IL-10 were determined by qPCR in IL-12 pretreated and untreated activated CD4 T cells stimulated with or without 6 μg/mL of plate bound anti-TCR antibodies (anti-CD3 clone OKT3) for 18 h. Data were normalized to that of mRNA encoding β-actin and presented relative to that of no cytokine cells. Results are shown as mean ± SEM of five separate donors. Data were analyzed with two-tail, unpaired Student’s t test. *p<0.05; **p<0.01; ***p<0.001; n.s. = not significant.

To further examine how IL-12 increases the global cytokine release, we examined the cells capable of responding to IL-12 from our heterogenous population of human activated CD4 T cells by determining the expression of IL-12 receptor before IL-12 pretreatment. IL-12 receptor is composed of two subunits IL-12R β1 and IL-12R β2. Co-expression of both subunits is required for the canonical cellular effects of IL-12 [9]. We found that IL-12R β1was highly upregulated and IL-12R β2 was minimally increased at the cell surface of activated CD4 T cells (Fig 3A). Then, the effect of IL-12 on TCR-mediated (anti-CD3 clone OKT3) cytokine expression was measured using intracellular staining. The TCR concentration dose used in these studies was 6 μg/mL since this was the lowest TCR dose that gave significant differences in the production of all cytokines. Exposure to IL-12 before TCR stimulation for 18h resulted in a significant increase in the frequency of cells producing IFN-γ and a slight, but statistically insignificant, increase in the frequency of cells producing TNF-α and IL-10 (Fig 3B and 3C). Surprisingly, the proportion of cells producing IL-4 and IL-13 remained the same between both IL-12 pretreated and control cells (Fig 3B and 3C). Furthermore, IL-12 pretreatment resulted in no differences in the MFI of IFN-γ, TNF-α, IL-4, IL-13 and IL-10 compared to untreated cells (Fig 3D). Interestingly, only a small fraction of the total cells used in our system (human activated CD4 T cells) responded potentially due to their expression of IL-12 receptor. The fact that IL-12 pretreatment increased the TCR-induced release of TNF-α, IL-4, IL-13 and IL-10 into the culture supernatants but had little to no effect when intracellular levels of these cytokines were measured using an inhibitor of protein transport from the endoplasmic reticulum to the Golgi apparatus (BFA) [40], suggests that IL-12 could mediate its effects by altering the release of cytokines. However because intracellular staining only provides a snapshot of cytokine production during a short time window, we confirmed our results by measuring intracellular cytokines at different time points and in the presence or absence of different secretion inhibitors (BFA and Monensin). We found a similar pattern of cytokine secretion when we repeated the experiments using BFA, Monensin, or no secretion inhibitor. Moreover, this cytokine secretion profile was observed at earlier and later time points of TCR induction (6h and 24h) (data not shown). Therefore, IL-12 increases the number of cells capable of producing IFN-γ, while altering the release of other cytokines via a separate mechanism.

### The IL-12 mediated enhancement of cytokine production following TCR stimulation is mediated by a function of both transcriptional and post-transcriptional effects

We next examined whether the IL-12 potentiation of cytokine production was a consequence of increased transcription of cytokines. To explore this possibility, total RNA was isolated from untreated and IL-12 treated human activated CD4 T cells before and after TCR ligation (anti-CD3 clone OKT3). Then the levels of mRNA expression of IFN-γ, TNF-α, IL-4, IL-13 and IL-10 were assessed by quantitative real-time PCR. In the absence of TCR stimulation there were no differences in the mRNA expression of the cytokines between IL-12 treated and untreated cells (Fig 3E), showing that IL-12 does not directly stimulate the production of cytokine mRNA. As expected, TCR induction upregulated the mRNA expression of IFN-γ, TNF-α, IL-4, IL-13 and IL-10 when compared to non-TCR stimulated cells (Fig 3E). TCR stimulation increased the mRNA expression of IFN-γ and lightly enhanced the mRNA expression of IL-10 in IL-12 pretreated cells in comparison to cells pretreated in media alone (Fig 3E). In contrast, the TCR-induced increase in TNF-α, IL-13 or IL-4 mRNA expression was not altered in IL-12-treated cells in comparison to untreated cells (Fig 3E). The same pattern was observed after shorter times of TCR induction (6–8 h), indicating that IL-12 pretreatment does not alter the kinetics of TCR-induced cytokine mRNA expression (data not shown). Together with the intracellular staining studies (Fig 3), these results suggest that the IL-12-mediated priming of cytokine release is driven by two separate mechanisms, increased mRNA expression for IFN-γ and increased release of TNF-α, IL-13, IL-4 and IL-10.

### IL-12 pretreatment enhances the activation of select signaling molecules downstream of the TCR

To further identify mechanisms by which IL-12 pretreatment potentiates the TCR-mediated production of cytokines, we examined the effects of IL-12 on the expression of surface molecules involved in T cell activation. We found no significant changes in the expression of the TCR, CD4, the costimulatory receptors CD28, ICOS or SLAM or the adhesion receptors CD2, CD49d, and CD11a after pretreatment with IL-12 (S4 Fig). This indicates that overt changes in surface expression of key receptors were not responsible for the effects of IL-12 on cytokine production.

**Fig 4:**
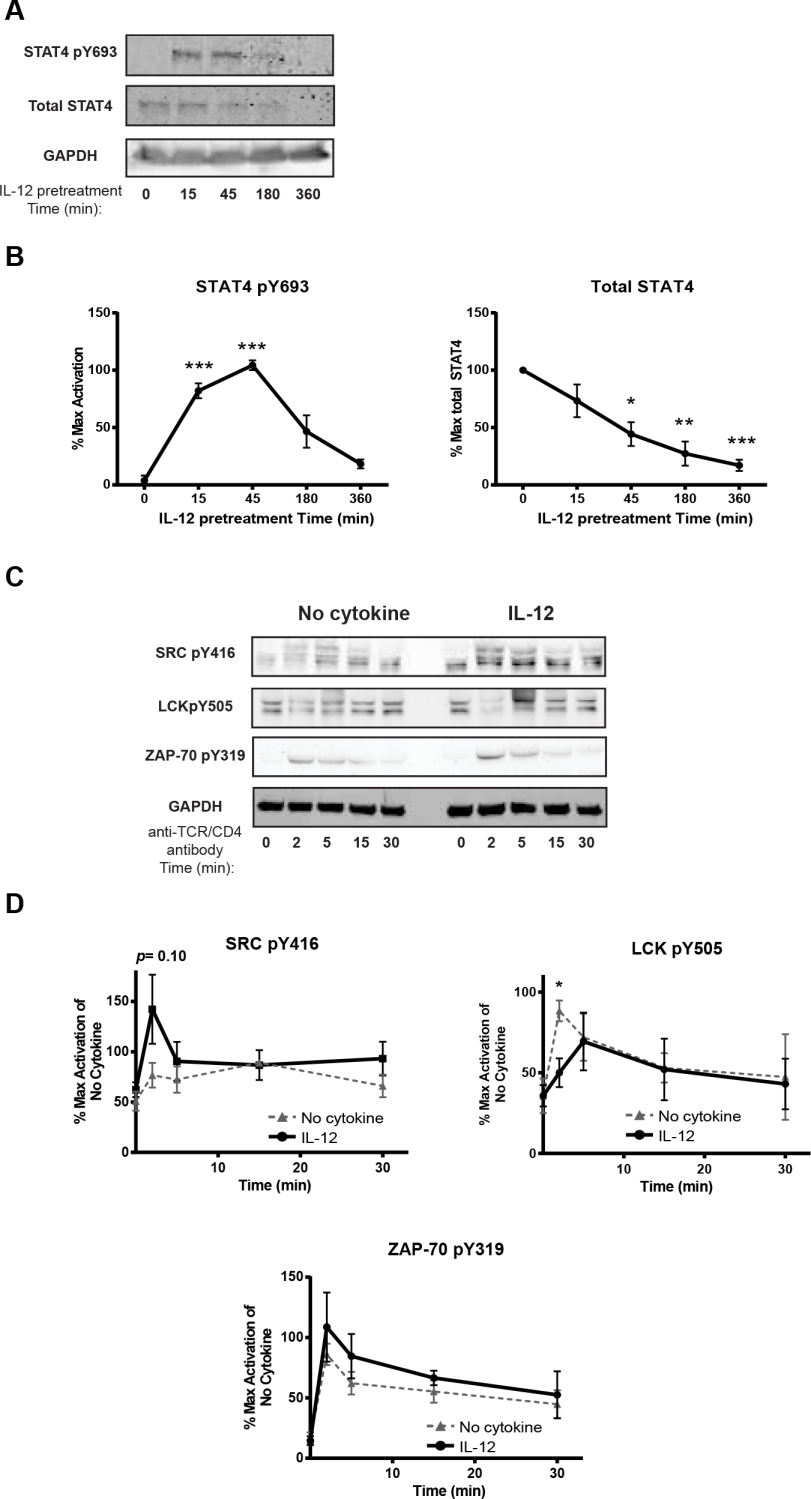
Prior exposure to IL-12 alters select TCR-induced signaling pathways in human activated CD4 T cells. Human activated CD4 T cells were (A and B) treated with or without IL-12 (50 ng/mL) for different times or (C and D) incubated with or without 50 ng/mL of IL-12 for 6 h and then stimulated with anti-TCR (anti-CD3 clone OKT3) and anti-CD4 (clone RPA-T4) antibodies for various times. The phosphorylation of signaling molecules was determined in whole cell lysates by immunoblotting. Results were normalized to GAPDH and the maximal level of activation of no cytokine cells. Data are shown as (A and C) representative blots from different donors and (B and D) mean ± SEM of normalized results of three to six separate donors. The loading controls shown for each figure correspond to at least one of the blots shown but for the quantification each blot was quantified with its respective control. Data were analyzed with two-tail, unpaired Student’s t test. *p<0.05; **p<0.01; ***p<0.001; n.s. = not significant.

**Fig 5:**
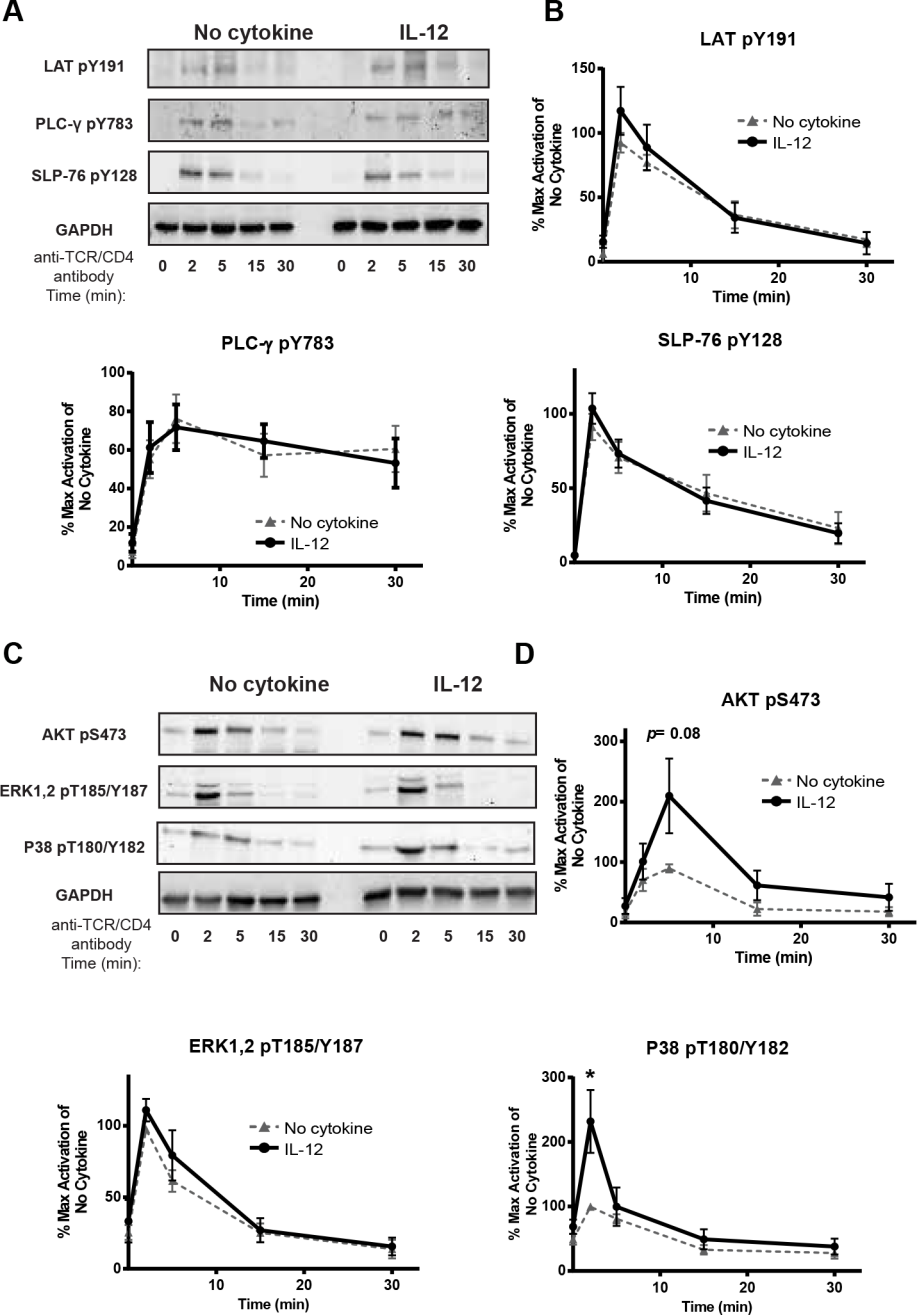
IL-12 pretreatment increases the activation of select signaling molecules downstream of the TCR. Human activated CD4 T cells were incubated with or without IL-12 (50 ng/mL for 6 h) and then stimulated with anti-TCR (anti-CD3 clone OKT3) and anti-CD4 (clone RPA-T4) for various times. The phosphorylation of signaling molecules was determined in whole cell lysates by immunoblotting. Results were normalized to GAPDH and the maximal level of activation of no cytokine cells. Data are shown as (A) and (C) representative blots from different donors and (B) and (D) mean ± SEM of normalized results of six to nine different donors. The loading controls shown for each figure correspond to at least one of the blots shown but for the quantification each blot was quantified with its respective control Data were analyzed with two-tail, unpaired Student’s t test. *p<0.05; **p<0.01; ***p<0.001; n.s. = not significant.

Following IL-12 stimulation, the Janus kinase-STAT signaling pathway is activated and leads to STAT4 phosphorylation. STAT4 is considered to be one of the critical mediators of the canonical IL-12 effects because STAT4-knockout mice have impaired Th1 differentiation and IFN-γ production [41]. Therefore, we examined if the IL-12-mediated increase of cytokine production following TCR stimulation was mediated directly by STAT4. To address this possibility, the total expression and the activation of STAT4 were determined before and after IL-12 pretreatment using quantitative immunoblotting. Treatment with IL-12 significantly increased the phosphorylation of STAT4 (Y693) peaking at 15–45 min following treatment. STAT4 phosphorylation returned back to near basal levels by the end of the six hour IL-12 pretreatment, which is the time when we stimulated the cells through the TCR in most of our experiments (Figs 4A and 5B). The total expression levels of STAT4 decreased significantly following IL-12 stimulation and became almost undetectable by the end of the IL-12 pretreatment, suggesting that STAT4 was getting degraded upon exposure to IL-12 signals (Figs 4A and 5B). Since STAT4 activation is similar before and after 6 hours of IL-12 pretreatment and STAT4 protein expression is almost undetectable after 6 hours of IL-12 pretreatment, we conclude that the IL-12 mediated increase in cytokine production upon TCR stimulation is likely not mediated directly by STAT4 alone synergizing with TCR stimulation signals.

Ligation of the TCR results in the activation of signaling molecules that ultimately promote transcriptional changes at cytokine genes. Therefore, we examined if IL-12 pretreatment altered the activation of signaling molecules downstream of the TCR. In support of this possibility, previous studies have shown that TCR-mediated signaling was enhanced in murine CD4 and CD8 T cells exposed to pathogen-induced inflammation [12, 13] and in human T cells pretreated with IL-7, IL-15 or the TLR5 ligand flagellin [15, 16]. To test this possibility, human activated CD4 T cells were left untreated or exposed to IL-12 and then stimulated using crosslinked anti-TCR/CD4 (anti-CD3 clone OKT3 and anti-CD4 clone RPA-T4) antibodies for various times. We use anti-TCR in combination with anti-CD4 for signaling studies because this provides the best enhancement of proximal and distal TCR signaling [20, 22, 23]. Then changes in the total expression and phosphorylation of TCR-induced signaling molecules were measured using quantitative immunoblotting. The activity of the tyrosine kinases LCK and ZAP-70 is induced early after TCR stimulation. LCK activation is dictated by the phosphorylation of Y394 and Y505, where increases in LCK Y394 and decreases in Y505 phosphorylation are correlated with enhanced LCK activity [42]. To detect changes in LCK Y394 phosphorylation, we used an anti-SRC pY416 antibody, which recognizes all SRC kinases, including LCK and FYN, when they are phosphorylated on their activating sites. LCK is the predominant kinase associated with CD4/plasma membrane and this kinase is the main phosphorylated SRC kinase detected in CD4 T cells [42]. Interestingly, IL-12 pretreatment resulted in a trend towards increased TCR-induced phosphorylation of LCK and FYN in comparison to cells treated in media alone; however this did not reach statistical significance (Fig 4C and 4D). In addition, IL-12 pretreatment significantly reduced the phosphorylation of LCK at its inhibitory site Y505 (Fig 4C and 4D). In contrast, the phosphorylation of the activating tyrosine on ZAP-70 (pY319) was similar between IL-12 treated and untreated groups (Fig 4C and 4D); the disconnection between LCK activity and ZAP-70 phosphorylation has been previously observed [21, 43].

ZAP-70 phosphorylates the adaptor protein LAT, resulting in the recruitment and phosphorylation of the phospholipase PLC-γ1 and adaptor protein SLP-76. Consistent with our previous data on ZAP-70, we observed no differences in the phosphorylation of LAT, SLP-76, or PLC-γ between control and IL-12 treated cells (Fig 5A and 5B). The recruitment and activation of proteins at LAT promotes the induction of the mitogen activated protein kinase (MAPK) and AKT pathways, resulting in the transcription of target genes that regulate cytokine production. We observed that IL-12 pretreated cells had a trend towards increased TCR-induced phosphorylation of AKT (S473) in comparison to control cells that did not reach statistical significance after compiling multiple donors (Fig 5C and 5D). Furthermore, we observed that the TCR-induced phosphorylation of p38 (pT180/T182) was significantly increased in IL-12 pretreated cells at 2 minutes in comparison to cells treated in media alone (Fig 5C and 5D). In contrast, there were no changes in the extent or kinetics of TCR-induced ERK1,2 (pT187/pY187) phosphorylation (Fig 5C and 5D). As shown in S4 Fig, the changes in activation of all the signaling molecules were not due to altered total expression of these proteins. To confirm its use as a loading control, we also examined the expression of GAPDH in both IL-12 pretreated and untreated human activated CD8 T cells in all the samples from the donors used in our studies. After quantifying the data of all the donors used in our studies, we found that both IL-12 pretreated and untreated cells have similar expression of GAPDH (S4 Fig). Overall, these findings suggest that prior exposure to IL-12 alters specific TCR-induced signaling pathways in human activated CD4 T cells.

### IL-12 mediated enhancement of cytokine secretion following TCR stimulation is partially regulated by an increase in oxidative metabolism

**Fig 6:**
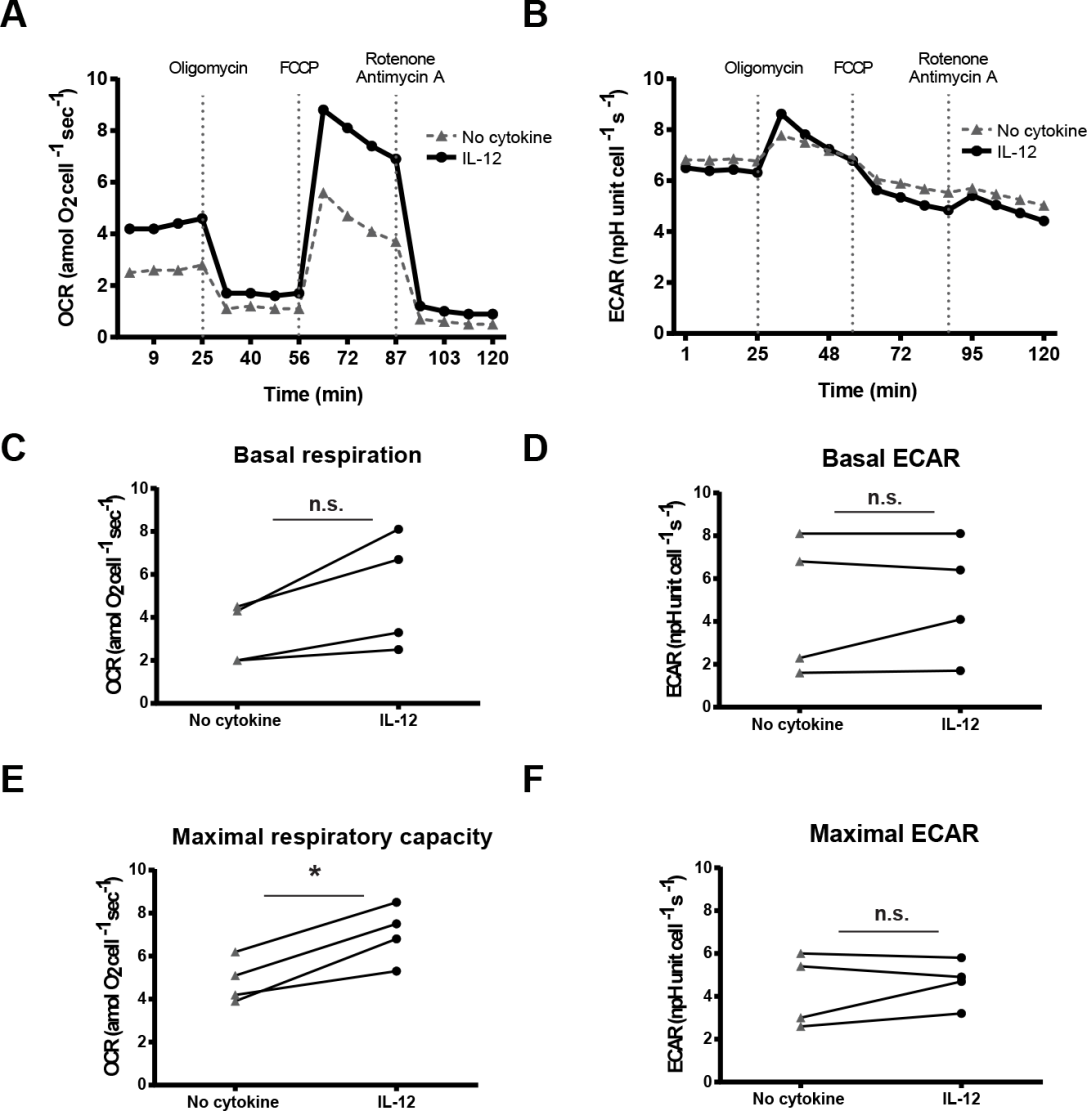
IL-12 pretreated human activated CD4 T cells undergo metabolic reprogramming towards oxidative metabolism. Human activated CD4 T cells were treated with or without 50 ng/mL IL-12 for 6 h. Cells were plated onto poly-l-lysine-coated plates and oxygen consumption rate (OCR) and extracellular acidification rate (ECAR) were assessed at basal state and following addition of indicated compounds. (A) and (B) graphs are representative of 4 different donors and used to calculate the (C) basal respiration, (D) basal ECAR, (E) maximal respiratory capacity, and (F) maximal ECAR. Data were analyzed with two-tail, unpaired Student’s t test. *p<0.05; **p<0.01; ***p<0.001; n.s. = not significant.

Our previous intracellular staining, mRNA expression, and signaling studies suggest that IL-12 pretreatment increases the release of cytokines *via* increased gene transcription and by an unknown post-transcriptional mechanism. Changes in the cellular function of immune cells, like cytokine secretion, can be post-transcriptionally regulated by alterations in metabolic pathways [44, 45]. This suggests that IL-12 exposure may alter the metabolic state of human activated CD4 T cells to drive the expression of cytokines regulated by a post-translational mechanism. To address this possibility, the metabolic profiles of IL-12 pretreated cells or untreated cells were determined using a metabolic flux analyzer. Mitochondrial respiration and aerobic glycolysis were assessed by measuring the oxygen consumption rate (OCR) and extracellular acidification rate (ECAR), respectively, under basal conditions and after drug-induced mitochondrial stress. Under basal conditions, IL-12 pretreated cells exhibited an increase in basal oxidative respiration compared to untreated cells as measured by the OCR (Fig 6A and 6C). The capacity of treated cells for oxidative metabolism was examined by measuring OCR after addition of oligomycin (an ATP synthase inhibitor that eliminates OCR due to ATP production), FCCP (a proton ionophore that uncouples ATP synthesis from the electron transport chain (ETC), which reveals the maximum capacity of the mitochondria to use oxidative metabolism), and rotenone plus antimycin A (an inhibitor of complexes I and III that completely shuts down the ETC). IL-12 pretreated cells exhibited a significant increase in the maximal respiratory capacity in comparison to cells treated in media alone (Fig 6A and 6E). This indicates that IL-12 treatment enhances mitochondrial respiration in human activated CD4 T cells. In contrast, basal and maximal ECAR, were similar in both IL-12 pretreated and untreated cells (Fig 6B and 6D and 6F). Finally, glucose consumption was determined in IL-12 pretreated and untreated cells using a fluorescent glucose analog (2-NBDG) that is used as an indicator for glucose uptake. Consistent with our previous data, we found that both IL-12 pretreated and untreated cells have similar 2-NBDG uptake (S6 Fig). Together, these findings suggest that IL-12 pretreated cells have enhanced oxidative metabolism, resulting in increased oxygen consumption rates and increased ability to up-regulate mitochondrial respiration in response to stimulation.

**Fig 7:**
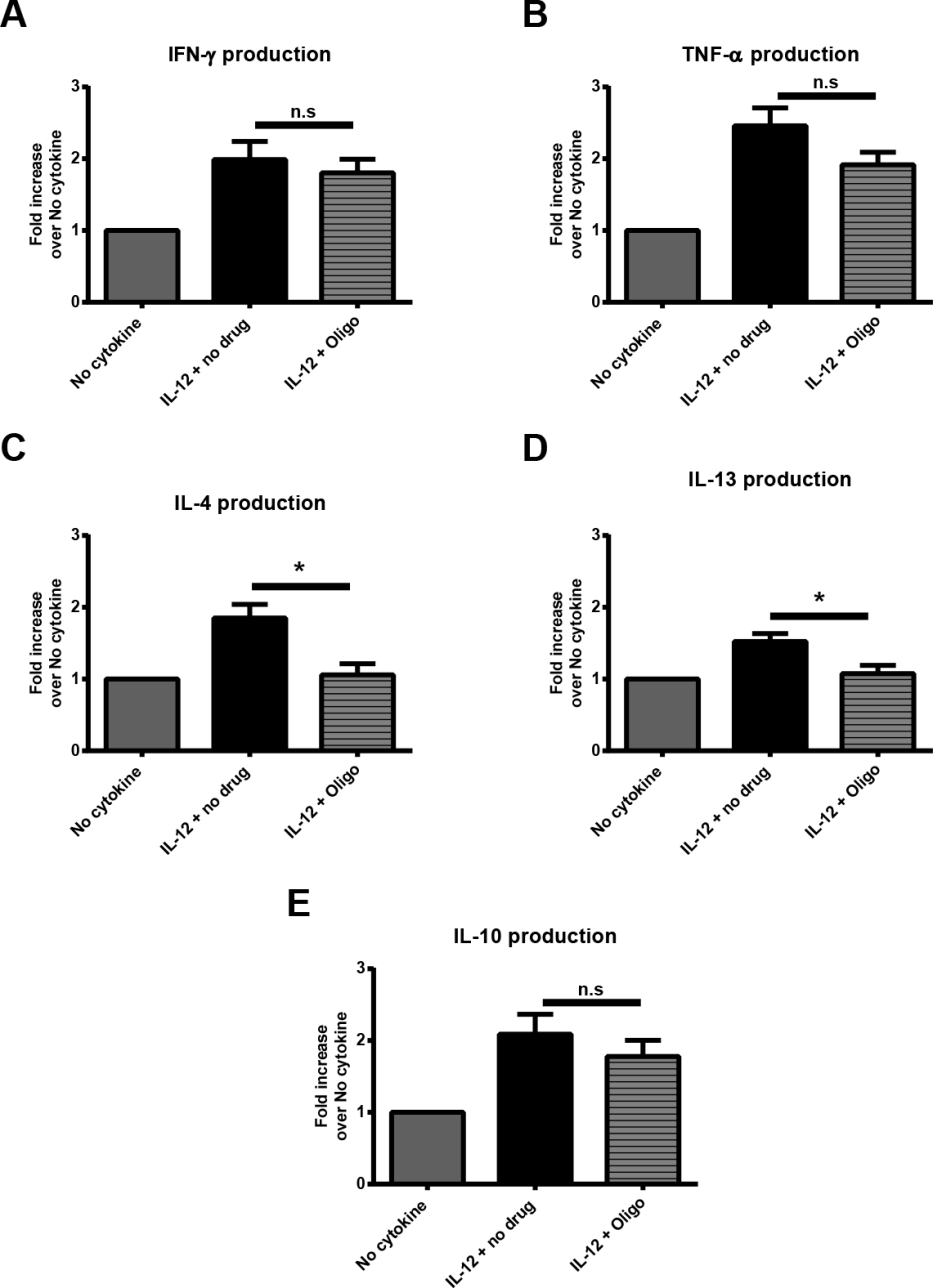
The IL-12-mediated enhancement of the secretion of cytokines not transcriptionally regulated is partially driven by an increase in oxidative metabolism. Human activated CD4 T cells were treated with or without 50 ng/mL of IL-12 for 6 h. Cells were then stimulated with 6 μg/mL plate bound anti-TCR antibodies (anti-CD3 clone OKT3) for 24 h in the presence or absence of oligomycin (2.5 μM). The protein levels of (A) IFN-γ, (B) TNF-α, (C) IL-4, (D) IL-13, and (E) IL-10 were determined by ELISA. Data for each treatment in an individual experiment were normalized to the amount produced by its respective control. The mean normalized value ± SEM from four to six different donors is shown. Data were analyzed with two-tail, unpaired Student’s t test. *p<0.05; **p<0.01; ***p<0.001; n.s. = not significant.

On the basis of these findings, we tested if the IL-12-mediated increase of oxidative metabolism was involved in regulating the priming of cytokines that were not transcriptionally regulated. To this end, IL-12 pretreated cells were TCR-stimulated (anti-CD3 clone OKT3) in the presence of an inhibitor of oxidative metabolism (oligomycin) and cytokine secretion was then measured. Consistent with published work, oligomycin was shown to effectively block mitochondrial respiration, as shown by the decrease of OCR in both IL-12 treated and untreated groups (Fig 6A). As expected, pretreatment with IL-12 resulted in the increase of IFN-γ, TNF-α, IL-4, IL-13, and IL-10 upon TCR stimulation in comparison to cells treated in media alone (Fig 7A–7E, S5 Fig). The IL-12 mediated enhancement of IFN-γ, TNF-α, and IL-10 was unaffected by the presence of oligomycin during TCR stimulation (Fig 7A, 7B and 7E, S5 Fig). However, unlike IL-12 pretreatment alone, pretreating with IL-12 in the presence of oligomycin did not augment TCR-induced IL-4 and IL-13 production (Fig 7C and 7D, S5 Fig). Notably, the dose of oligomycin used had minimal effects on the production of cytokines by the cells treated in media alone (S5 Fig). In addition, we also tested the role of glucose metabolism in the TCR-induced production of cytokines in both untreated and IL-12 pretreated cells. To this end, IL-12 pretreated cells were TCR-stimulated in the presence of an inhibitor of glycolysis (2-DG) and cytokine secretion was then measured. We found that cells treated both with media alone or IL-12, 2-DG significantly decreased the TCR-induced production of IFN-γ, TNF-α, IL-4, and IL-10 (S6 Fig). These data is consistent with a previous study that examined the effects of 2-DG in the TCR-induced production of cytokines in human T cells and suggest that glycolysis is critical for TCR-mediated cytokine production [46]. These findings suggest that IL-12-mediated increase in oxidative metabolism is involved in regulating the release of IL-4 and IL-13 without altering the release of IFN-γ, TNF-α, and IL-10.

## Discussion

Previous studies from us and others has demonstrated that prior exposure of activated T cells to inflammatory signals increases the responsiveness of these cells to subsequent TCR stimulation. For example, pretreatment with IL-7, IL-15 or a TLR5 ligand increases the responsiveness of human T cells to TCR stimulation [15, 16]. In addition, murine effector/memory CD8 or secondary effector CD4 T cells exposed to pathogen induced-inflammation have enhanced ability to respond to TCR stimulation [12, 13]. Similarly, murine memory CD8 T cells conditioned with IL-12 and IL-18 *in vitro* have enhanced cytokine production and cytotoxic activity upon TCR re-stimulation [14]. Herein, we demonstrate that prior exposure of human activated CD4 T cells to IL-12 results in increased production of a range of cytokines upon TCR re-stimulation. Mechanistically, we observed that IL-12 mediated its effects through both transcriptional and post-transcriptional mechanisms. The IL-12-driven priming of IFN-γ production was linked to increased mRNA expression, likely mediated by the enhanced activation of certain TCR signaling molecules. In contrast, the IL-12-mediated enhancement of cytokines that are not transcriptionally regulated was partially driven by increased oxidative metabolism. Collectively, these studies highlight underappreciated roles for inflammatory signals in continually shaping TCR-mediated responses via context-specific mechanisms.

The canonical role for IL-12 in naïve CD4 T cells is the differentiation of Th1 cells. Based on this model, we expected IL-12 pretreatment to induce primarily IFN-γ and TNF-α production upon TCR stimulation [39]. To our surprise, IL-12 pretreatment of human activated CD4 T cells led to a general upregulation of cytokine production upon TCR stimulation. Interestingly, the effects of IL-12 pretreatment in enhancing the production of IL-4 and IL-13 were not as robust as for other cytokines. However, the levels of IL-4 and IL-13 produced in our studies are similar to what it has been observed in human ex-vivo isolated Th2 cells [2, 47]. After polarization, human CD4 T cells remain flexible and can be re-polarized in different inflammatory environments [2, 3]. Furthermore, emerging literature suggests that full CD4 T cell differentiation occurs at sites of infection and inflammation after they have migrated from the secondary lymphoid organs [1]. Therefore, beyond its role in Th1 polarization, IL-12 may also prepare primed, but not fully differentiated, effector CD4 T cells to release numerous cytokines prior to their final polarization. Similar to our study, the IL-12-mediated production of cytokines other than IFN-γ has been previous reported in T clones, peripheral blood T cells, and tumor reactive T cells [48–52]. Likewise, several preclinical studies have shown that IL-12 administration increases plasma levels of IFN-γ and other cytokines, such as IL-10 [53–55]. Our data fits well with these findings and demonstrates that the regulation of CD4 T cells responses by IL-12 is more complex than previously appreciated.

The effects of IL-12 on human activated CD4 T cells had several interesting features. First, short-term exposure to different doses of IL-12 was sufficient to transiently alter responses to TCR stimulation. The fact that the IL-12 mediated effects on human activated CD4 T cells were short lived suggests that the priming of activated CD4 T cells is tightly regulated to minimize the risk of immunopathology. Next, pretreatment of human activated CD4 T cells with IL-12 did not enhance TCR functional avidity, at least using an antibody based system to stimulate the TCR. Interestingly, previous studies on murine CD8 and CD4 T cells, using an antigen based system found that IL-12 signals from pathogen induced inflammation enhanced the functional avidity of these cells [12, 13]. These distinctions could be attributed to differences between human and mouse T cell responses or caused by experimental variations in our defined IL-12 treatment versus *in vivo* inflammation driven by many cytokines. Finally, the proportion of cells making TNF-α, IL-10, IL-4 and IL-13, and the MFI of all assessed cytokines was not significantly altered by IL-12 pretreatment. We believe that by using an inhibitor of protein transport from the endoplasmic reticulum to the Golgi apparatus [40], we were incapable of detecting IL-12-driven effects in cytokine release downstream of the inhibitor. Similarly, IL-12 pretreatment altered the gene expression of IFN-γ, but not TNF-α, IL-4, IL-13 or IL-10, following TCR stimulation. Together, these data suggest that IL-12 mediates its effects by at least two mechanisms: increased mRNA expression of IFN-γ resulting in more cells releasing this cytokine and increased ability of cells to release TNF-α, IL-4, IL-13 and IL-10.

We have begun to characterize the molecular mechanism by which IL-12 potentiates the TCR-mediated production of a range of cytokines. We found that IL-12 exposure significantly increased the TCR induced activation of p38 and resulted in a trend towards increased activation of AKT without altering the activation of other signaling molecules. IL-12 pretreatment also seems to increase the TCR activation of LCK as shown by the a trend towards increased TCR induced phosphorylation of the activating sites in LCK and FYN and a significant reduced phosphorylation of LCK at its inhibitory site Y505. In contrast, pathogen-induced inflammation increased proximal TCR signaling (ZAP-70, PLC-γ) and ERK1/2 and JNK1/2 without altering the activation of p38 in murine effector/memory CD8 T cells and increased ZAP-70 and ERK1/2 in murine secondary effector CD4 T cells [12, 13]. Interestingly, in human T cells IL-7 and IL-15 mediated their effects by increasing the activation of ERK1/2 following TCR stimulation [16]. Our laboratory also observed that prior activation of T cells with a TLR5 ligand enhances TCR-mediated AKT activation, while simultaneously reducing LCK and LAT phosphorylation [15]. These studies suggest that each inflammatory signal potentiates TCR-mediated signaling via a distinct molecular mechanism.

A previous report has shown that p38 plays an important role in the production of IFN-γ by murine Th1 CD4 T cells *in vitro*. In this study, the authors demonstrate that blockade of p38 activity inhibits the gene expression of IFN-γ [56]. Furthermore, AKT activation has been shown to have multiple roles on T cell responses including cytokine release [57]. Unpublished data from our laboratory suggests that the presence of an AKT inhibitor (BML 257) reduces TCR-mediated IL-2 production, indicating the critical role that this kinase plays in cytokine production in human T cells. Similar to our findings, previous literature has reported a crosstalk between IL-12 signals and LCK, AKT, and p38. Visconti and colleagues demonstrated that IL-12 signals activate p38 and AKT pathways without altering ERK or JNK activation in T cells [58]. Furthermore, in human resting and activated NK cells, IL-12 signals were shown to increase LCK activation. Future studies will have to explore how IL-12 signals alter the activation of LCK, AKT, and p38 following TCR stimulation without altering more proximal TCR signals. Together, our results suggest that the IL-12-mediated increase of p38 and AKT could be responsible for the increased gene expression of IFN-γ. This correlation will be further examined in future studies to determine if the IL-12 mediated changes in TCR signaling are in fact responsible for the increased gene expression of IFN-γ.

We also found that IL-12 pretreated cells have increased oxidative metabolism which partially regulates the release of IL-4 and IL-13. This suggests that the IL-12 enhancement of cytokines that are not transcriptionally regulated could be partially attributed to enhanced oxidative metabolism. This seems to be a particular effect of IL-12, since exposure to other inflammatory cytokines like TNF-α did not alter metabolic pathways (data not shown). Previous reports have demonstrated that brief exposure to inflammatory signals alters the metabolic state of different immune cells [59–62]. Furthermore, previous reports have demonstrated that changes in metabolic pathways can regulate immune cellular function like cytokine secretion and proliferation. For T cells, the production of IFN-γ is post-transcriptionally controlled by aerobic glycolysis [44]. Similarly, for DCs, the LPS-induced production of cytokines IL-6, IL-12 and TNF-α is also regulated at the translational level by aerobic glycolysis [45]. However, our studies are the first to demonstrate that the cytokine IL-12 promotes metabolic reprograming towards oxidative metabolism, and that oxidative metabolism is involved in the regulation of cellular functions like cytokine secretion.

Our findings highlight a critical question: what is the physiological role of the potentiation of cytokine release by IL-12? Naive CD4 T cells are primed and activated in secondary lymphoid tissues. These cells then migrate through the circulatory or lymphatic system before entering into the sites of infection. During this migration, these cells are exposed to IL-12 and other inflammatory signals. Since human CD4 T cells remain capable of differentiating into multiple CD4 T cell lineages after initial priming and activation, we propose a model in which IL-12 present in blood, infection sites, and/or at inflammatory sites alters human activated CD4 T cells that have not terminally differentiated, rendering them capable of producing a range of cytokines upon TCR activation. This allows the cells to respond faster when they encounter their cognate antigen at sites of infection and be easily polarized into CD4 helper T cell subsets depending upon the cytokine milieu they encounter. This function of IL-12 is the likely reason why clinical trials of IL-12 to treat human cancer have shown limited efficacy [54, 55]. IL-12 treatment will not only drive Th1 T cell differentiation, but also enhance the production of Th2 cytokines and suppressive cytokines such as IL-10. In fact, IL-10 levels are increased in patients treated with IL-12 [54, 55].

In conclusion, our studies have uncovered that, beyond its role in Th1 differentiation, IL-12 elevates the responses of activated CD4 T cells for further TCR stimulation by altering TCR signaling pathways and by increasing metabolic respiration. Our findings increase our understanding of the physiologic properties of human CD4 T cells and provide insights into potential avenues to improve the current uses of IL-12 in therapeutics.

## Supporting Information

S1 Fig Characterizing the effects of prior IL-12 exposure on subsequent TCR induced IFN-γ production. Human activated CD4 T cells were left untreated (no cytokine) or treated for 6 h with various cytokines (50 ng/mL) (A). Alternatively, cells were treated for 6 h with various doses of IL-12 (B), treated for various times with 50 ng/mL of IL-12 (C), or treated for 6 h with 50 ng/mL of IL-12 (D). After pretreatment, cells were washed and stimulated with 2 μg/mL of plate bound anti-TCR antibodies for 24 h (A-C). Alternatively, pretreated cells were rested for various times and then stimulated with plate bound anti-TCR antibodies (2 μg/mL) for 24 h (D). IFN-γ production was determined by ELISA. The mean ± SEM values from four to seven different donors are shown.
(EPS)

S2 Fig IL-12 pretreatment did not alter the proliferation/survival or expression of CD25. (A) Human activated CD4 T cells were incubated with or without various cytokines (50 ng/mL for 6 h), washed, and stimulated with 2 μg/mL of plate bound anti-TCR antibodies for 24 h. Viable cell numbers were determined by using the trypan blue dye exclusion assay. Graphs show the mean ± SEM values from five separate donors. Data were statistically compared to cells treated in media alone (no cytokine) with a two-tail, unpaired Student’s t test. *p<0.05; **p<0.01; ***p<0.001; n.s. = not significant. (B-C) Human activated CD4 T cells were left untreated or pretreated with 50 ng/mL of IL-12 for 6 h, washed, and then stained with annexin V in a buffer containing propidium iodine before and after TCR stimulation. Cells were then analyzed by flow cytometry. Data are shown as representative plots of two donors. (D) The expression of CD25 was examined in human activated CD4 T cells pretreated or untreated with IL-12 (50 ng/mL for 6h) by flow cytometry. Dotted gray line represent unstained control, gray line represents CD25 staining of untreated cells, and black line represents CD25 staining of IL-12 pretreated cells. Blots are representative of two donors.
(EPS)

S3 Fig Prior exposure to IL-12 enhances cytokine production following TCR stimulation. Human activated CD4 T cells were left untreated or pretreated with 50 ng/mL of IL-12 for 6 h and then subsequently stimulated with various doses of plate bound anti-TCR antibodies for 24 h. Protein levels of IFN-γ, TNF-α, IL-4, IL-13, and IL-10 were assessed by ELISA. The mean value ± SEM from four to five different donors is shown.
(EPS)

S4 Fig IL-12 pretreatment does not alter the expression of surface molecules or TCR signaling molecules. Human activated CD4 T cells were incubated with or without IL-12 (50 ng/mL for 6 h). In (A) changes on the expression of surface molecules were assessed by flow cytometry. Cells were gated on live cells and then the MFIs were determined. The mean value ± SEM from three to four different donors is shown. In (B), pretreated cells were stimulated with anti-TCR and anti-CD4 antibodies for various times. The total expression of signaling molecules were determined in whole cell lysates by immunoblotting and the results were normalized to GAPDH. Data are shown as mean value ± SEM of two to six different donors. In (C) the expression of GAPDH was assessed in whole cell lysates by immunoblotting. Data are shown as mean value ± SEM of twenty different donors. Results were analyzed with two-tail, unpaired Student’s t test. *p<0.05; **p<0.01; ***p<0.001; n.s. = not significant.
(EPS)

S5 Fig The IL-12 mediated enhancement of cytokine secretion following TCR stimulation is partially regulated by an increase in oxidative metabolism. Human activated CD4 T cells were left untreated or pretreated with 50 ng/mL of IL-12 for 6 h. Cells were then subsequently stimulated with 6 μg/mL plate bound anti-TCR antibodies for 24 h in the presence or absence of oligomycin (2.5 μM). The protein levels of IFN-γ, TNF-α, IL-4, IL-13, and IL-10 were determined by ELISA. The mean value ±SEM from four to six different donors is shown.
(EPS)

S6 Fig Glucose consumption and the effects of glycolysis inhibition on IL-12 pretreated and untreated cells. Human activated CD4 T cells were left untreated or pretreated with 50 ng/mL of IL-12 for 6 h. In (A) glucose consumption was assessed using a fluorescent glucose analog that is used as an indicator for glucose uptake (2-NBDG). Dotted gray line represent unstained control, gray line represents untreated cells, and black line represents IL-12 pretreated cells. Blot is representative of two donors. In (B), IL-12 pretreated or untreated cells were stimulated with 6 μg/mL plate bound anti-TCR antibodies for 24 h in the presence or absence of 2-DG (5 mM). The protein levels of IFN-γ, TNF-α, IL-4, and IL-10 were determined by ELISA. The mean value ±SEM from two different donors is shown.
(EPS)
